# Associations between vitamin D levels and dietary patterns in patients with Hashimoto’s thyroiditis

**DOI:** 10.3389/fnut.2023.1188612

**Published:** 2023-05-05

**Authors:** Dean Kaličanin, Maja Cvek, Ana Barić, Veselin Škrabić, Ante Punda, Vesna Boraska Perica

**Affiliations:** ^1^Department of Medical Biology, University of Split School of Medicine, Split, Croatia; ^2^Department of Nuclear Medicine, University Hospital of Split, Split, Croatia; ^3^Department of Paediatrics, University Hospital of Split, Split, Croatia

**Keywords:** autoimmune thyroid disease, coffee intake, 25 hydroxy vitamin D, sweets intake, vegetables intake, food frequency questionnaire

## Abstract

**Introduction:**

Vitamin D insufficiency is a global health problem affecting healthy and diseased individuals, including patients with Hashimoto’s thyroiditis (HT). Identifying dietary factors that may affect vitamin D levels and providing dietary guidelines accordingly can alleviate this problem. We therefore aimed to identify still unknown associations of dietary patterns, assessed through the Food Frequency Questionnaire (FFQ) with vitamin D blood levels.

**Materials and methods:**

FFQ was collected from 459 patients from Croatian Biobank of Patients with Hashimoto’s thyroiditis (CROHT), while total 25(OH)D was measured from their stored serum samples. We performed linear regression analysis between vitamin D levels and weekly intake of 24 food groups in 459 patients with HT (ALL), and in two disease-severity groups (MILD and OVERT).

**Results:**

The main results of our study are observations of: (1) an inverse association between vitamin D levels and coffee consumption (ALL: *β* = −0.433, *p* = 0.005; OVERT: *β* = −0.62, *p* = 0.008); (2) an inverse association between vitamin D levels and sweets consumption (ALL: *β* = −0.195, *p* = 0.034; OVERT: *β* = −0.431, *p* = 0.006); (3) positive association between vitamin D levels and vegetable consumption (ALL: *β* = 0.182, *p* = 0.019; OVERT, *β* = 0.311, *p* = 0.009). Importantly, effect sizes of all three associations were more prominent in HT patients with prolonged and more severe disease (OVERT).

**Conclusion:**

Further research into the functional and causal relationships of the observed associations is important to provide guidance regarding coffee/sugar intake on vitamin D status. A well-balanced diet can help prevent vitamin D deficiency and improve the quality of life of patients with HT, especially those in later stages of disease characterized by greater metabolic imbalance.

## 1. Introduction

Hashimoto’s thyroiditis (HT) is considered the most common thyroid disorder and one of the most frequent autoimmune disorders in general (1). It has constant rise in incidence and it mostly affects female population, especially women between the ages of 30 and 60 (2, 3). The main characteristic of HT is damage of thyroid tissue resulting from lymphocytic infiltration that usually leads to hypothyroidism (4). For that reason, HT is the major cause of hypothyroidism in developed areas of the world (2). Another important characteristics of HT is the presence of thyroid antibodies against thyroid peroxidase (TPOAb) and/or thyroglobulin (TgAb), which are key markers for diagnosis of HT, along with a characteristic thyroid ultrasound (5).

Genetic, environmental and existential factors play a role in the etiology of HT (6). Environmental factors trigger the disease onset and high iodine intake is considered the main factor in the development of HT, while several other proposed risk factors include viral and bacterial infections, intestinal microbiota, low selenium, iron deficiency, various toxins, and drugs (6–9). In addition, dietary factors have also been suggested to act as environmental modifiers of disease (10–16). In the last decade, vitamin D insufficiency/deficiency has been associated with various endocrine and autoimmune diseases (diabetes, systemic lupus erythematosus, multiple sclerosis, rheumatoid arthritis, autoimmune thyroid disorders (AITD), adrenal diseases, cardiovascular diseases), cancers, infections, inflammatory responses and depression (17). Our research group has already analyzed associations of dietary habits with HT (10) and vitamin D levels with HT (18). In this paper, we focus on the analysis of dietary patterns associated to vitamin D levels in patients with HT.

Vitamin D is a steroid prohormone with confirmed role in calcium and phosphorus homeostasis and regulation of bone mineral metabolism. Biologically active form of vitamin D, calcitriol (1,25(OH)2D) exerts its role by binding to the nuclear vitamin D receptor (VDR) (19) which then regulates the expression of more than 200 genes (3–5% of the human genome) (20). The main circulating form of vitamin D, calcidiol 25(OH)D, has been established as an main indicator of vitamin D sufficiency in the body (21). Vitamin D sufficiency levels in Croatia are the same as those recommended by The American Endocrinology Society: a sufficiency stands for 25(OH)D levels ≥30 ng/mL (75 nmol/L), insufficiency is defined for levels between 21 AND 29 ng/mL (50–75 nmol/L) whereas deficiency is considered when 25(OH)D levels ≤20 ng/mL (50 nmol/L). Although, some other guidelines propose lower vitamin D sufficiency threshold of 20 ng/mL (22), we follow guidelines that are valid in our population.

Humans get vitamin D from three different sources: sun exposure, dietary intake and supplementation. It is estimated that about 80% of the total vitamin D is in the form of vitamin D3 which is mainly produced in skin from 7-dehydrocholecalciferol, under exposure to sunlight. Smaller content comes from food of animal origin such as fish (wild fresh salmon, sardines, cod liver oil), egg yolk, fortified milk, cheese, and meat (beef, pork) (23–25). Milk is fortified with vitamin D in the United States and Canada, however, majority of European countries do not fortify milk with vitamin D. Vitamin D2 is not synthesized in the body, but can be taken in with food of plant origin (mushrooms, yeast) (24). The production of vitamin D is not stable, so the produced amount depends on: sex, age, body mass index (BMI), skin pigmentation, latitudes, season, number of sunny days, weather, sun exposure, use of sunscreen, time spent indoor, sedentary lifestyle, cultural and religious habits and customs, education, dietary behavior, (in)availability of food rich in vitamin D (26–28).

Many studies observed positive effects of intake of fortified food or food rich in vitamin D (eggs and fatty fish) on blood vitamin D levels (23, 29–32). Adherence to the Mediterranean diet, characterized by high consumption of polyphenols, fibers and monounsaturated fatty acids (MUFA) (33), is also associated with elevated vitamin D levels (34).

Our study aims to comprehensively analyze associations of dietary habits, assessed through the food frequency questionnaire (FFQ), and vitamin D levels in a group of patients with HT from the Croatian Biobank of HT patients (CROHT biobank) (35, 36). To consider the influence of HT-disease severity on relationship between food intake and vitamin D levels, we also performed analyses in two subgroups of HT patients, those that are in the early stages of HT (MILD) and those in progressed HT (OVERT). To our knowledge, this is the first study that analyzed associations of dietary factors/patterns and vitamin D levels in patients with the most common autoimmune thyroid disorder.

## 2. Materials and methods

### 2.1. CROHT biobank

**Figure 1:**
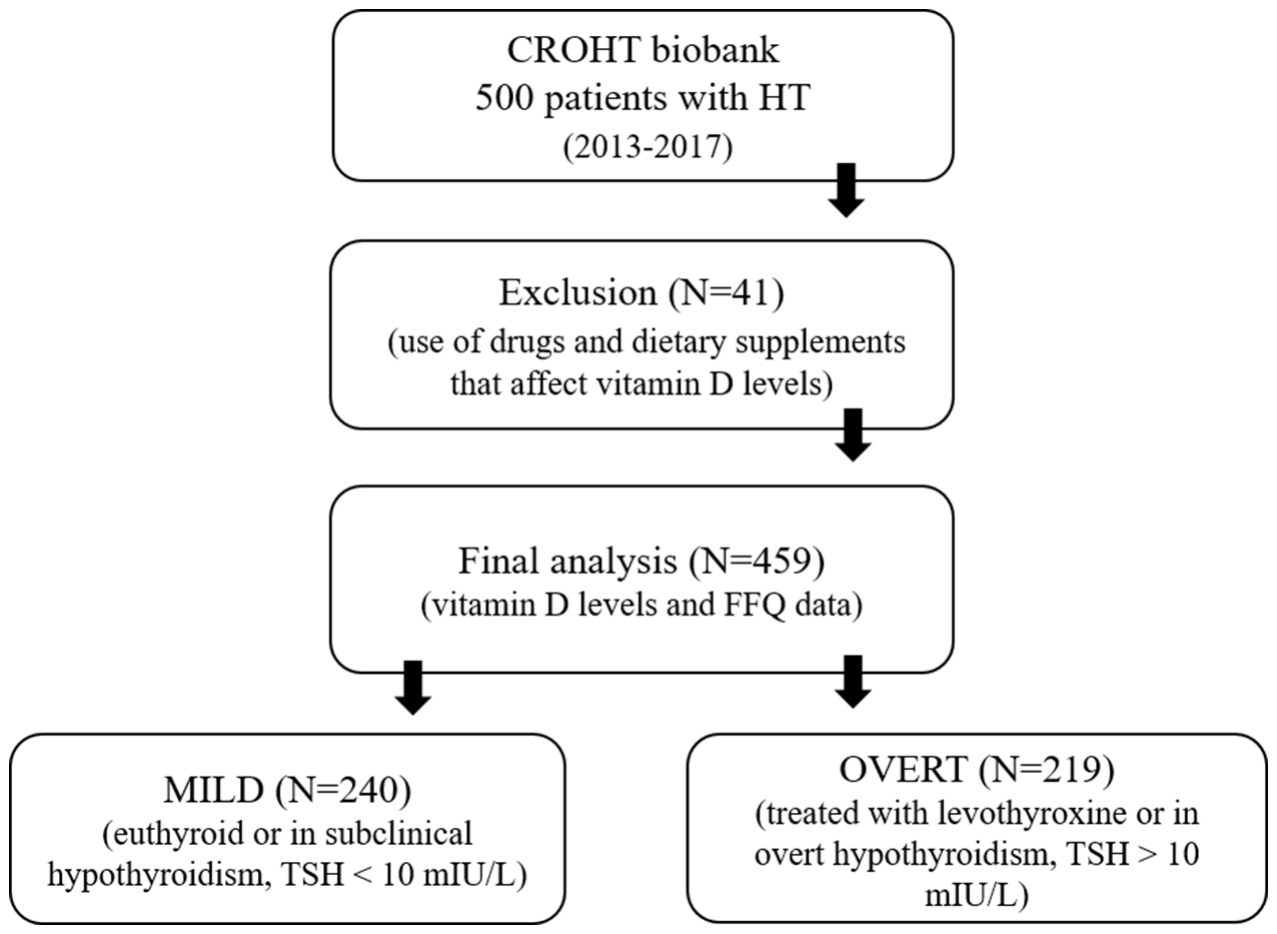
Recruitment of patients with Hashimoto’s thyroiditis (HT) in the study.

HT patients were derived from the recently established CROHT biobank (36). The CROHT biobank contains stored biological samples (DNA, serum and plasma), data of more than 200 phenotypes and various clinical measures for 500 HT patients. Patient recruitment was carried out in the Outpatient clinic for thyroid disorders in the Clinical department of nuclear medicine at the University Hospital of Split from 2013 to 2017 (Figure 1). Diagnosis of HT was determined by the specialists in nuclear medicine, following European Thyroid Association (ETA) recommendations and guidelines for the Management of Subclinical Hypothyroidism (37). In more detail, HT was diagnosed using data obtained by clinical examination, thyroid ultrasound (echographic pattern of diffuse thyroid disease) and assessment of thyroid-related biochemical parameters (increased thyroid-stimulating hormone (TSH) and/or decreased thyroid hormones: triiodothyronine (T3), thyroxine (T4), or free thyroxine (fT4) and/or increased thyroid autoantibodies (TPOAb and TgAb)). Reference ranges for our population are: TSH (0.3–3.6 mIU/L), T3 (1.3–3.6 nmol/L), T4 (57.3–161 nmol/L), fT4 (10.3–22.8 pmol/l), TPOAb (1–16 IU/mL), TgAb (5–100 IU/mL). Plasma levels of TSH, T3, T4, fT4, TgAb, and TPOAb were measured by immunoassay using the fully automated “Liaison” Biomedica Chemiluminescence Analyzer (DiaSorin, Saluggia, Italy).

Total 25(OH)D (vitamin D in the text) was measured from the stored serum samples in 459 HT patients (92.37% of females), using LIAISON 25(OH) Vitamin D Total chemiluminescence immunoassay (DiaSorin, Saluggia, Italy). Recruitment of HT patients took place uniformly throughout all four seasons of the year. CROHT biobank was thoroughly searched, for each HT patient, for the use of drugs and dietary supplements that affect vitamin D levels, such as corticosteroids, anticonvulsants, vitamin D supplements or daily use of calcium supplements. HT patients (*N* = 41) using any of these medications/supplementations were excluded from this study.

Prior to recruitment to CROHT biobank all HT patients: (i) were introduced to general aims of scientific projects performed under CROHT biobank; (ii) gave consent for usage of their samples for future scientific research and (iii) signed an Agreement for participation. Ethics Committee from the University of Split School of Medicine (Classification no. 003–08/14–03/0001 and Registry no. 2181–198–03-04-14-0028; Classification no. 003–08/19–03/0003 and Reg. no. 2181–198–03-04-19-0019) and the Ethics Committee from the University Hospital of Split (Classification no. 530–02/13–01/11; Registry no. 2181–147-01/06/J.B.-14–2; Classification no. 500–03/18–01/80 and Reg. no. 2181–147-01/06/M.S.-18–2) approved this research and declared that it was in accordance with the provisions of the Code of Ethics and the Helsinki Declaration.

### 2.2. Assessment of dietary intake

The dietary habits of patients with HT were collected through FFQ developed by experienced researchers and used in a previous study (10). The FFQ assessed the frequency of consumption of 51 food products, using categories: every day/always (converted to 7), 2–3 times a week (converted to 2.5), once a week/sometimes (converted to 1), once a month (converted to 0.25), rarely (converted to 0.125) and never (converted to 0). Plant oil, olive oil and animal fat were assessed using additional question with three consumption frequencies: always, sometimes, and never. We calculated weekly consumption of 54 food items and grouped them into 24 food categories.

### 2.3. Statistical analysis

Continuous variables were presented as mean and standard deviation (SD) or median and first- third quartile (Q1–Q3). Differences between tested groups were assessed with Mann Whitney U-test and *t*-test for independent samples. Categorical variables were presented as whole numbers and percentages, while differences were assessed with the chi-squared test. The association between vitamin D levels and weekly intake of 24 food groups was examined using linear regression model, where vitamin D level was the dependent variable and 24 food groups were independent variables. We performed linear regression analysis in all 459 patients with HT (ALL), and in the two subgroups depending on HT severity at the time of recruitment (MILD and OVERT). Patients with HT who were euthyroid (TSH within reference ranges) or in subclinical hypothyroidism (TSH within the range of 3.6–10 mIU/L) were assigned to the MILD group. Patients who were in overt hypothyroidism (TSH > 10 mIU/L) or were treated with levothyroxine (LT4) therapy were assigned to the OVERT group.

We included age, sex, score of physical activity, body mass index (BMI), smoking status and seasonality of blood sampling as covariates in each model. Additionally, we included LT4 therapy status as a covariate when performing analyses in ALL and OVERT groups. Kolmogorov–Smirnov test was used for testing normality of residuals distribution while Levene’s test was used for testing homogeneity of variance. Statistical analyses were performed using SPSS statistical software (SPSS Inc., Chicago, IL, United States).

## 3. Results

The main clinical and sociodemographic characteristic of ALL 459 patients with HT and two subgroups of patients with HT according to disease severity (240 MILD and 219 OVERT) are presented in Table 1. We observed high proportions of vitamin D deficiency and insufficiency in our cohort. More precisely, vitamin D deficiency was observed in 51.84% of ALL, 47.92% of MILD and 56.16% of OVERT HT patients. Strikingly, very small proportion of participants had sufficient vitamin D levels: 10.63% in ALL, 11.66% in MILD and 9.59% in OVERT. Regarding other clinical information, the two subgroups of patients have comparable levels of TSH and thyroid hormones (Table 1), however, the thyroid gland of patients in the early stage of the disease (MILD) is still able to produce sufficient amounts of hormones, whereas the thyroid gland of patients from the OVERT group is no longer functioning adequately and euthyroidism is mostly restored due to synthetic hormone intake (LT4 therapy). Other clinical features indicate that patients from the OVERT group are in more advanced and severe stage of HT compared to patients from the MILD group as they have higher median values for: BMI, anti-thyroid antibodies and the number of hypothyroidism symptoms. It is very useful to have two groups of patients that reflect the severity of HT as it enables us to investigate how/if the clinical characteristic in question changes with disease exacerbation.

**Table 1:** Clinical characteristics of HT patients (ALL) and HT patients divided in two disease-severity groups (MILD and OVERT).

Phenotype	ALL	MILD	OVERT	P*
	(*N* = 459)	(*N* = 240)	(*N* = 219)	
	Median (Q1–Q3)	Median (Q1–Q3)	Median (Q1–Q3)	
Female, N (%)	424 (92.37)	226 (94.17)	198 (90.41)	0.129
Age, years	38.02 (27.76–48.49)	35.81 (25.78–46.95)	40.28 (31.04–50.37)	0.002
BMI, kg/m^2^	23.52 (20.76–26.85)	23.15 (20.72–26.59)	24.01 (21.02–26.99)	0.043
Vitamin D, ng/mL	19.70 (14.40–25.20)	20.70 (14.90–25.80)	19.00 (13.95–24.55)	0.055
Vitamin D deficiency, N (%)	238 (51.84)	115 (47.92)	123 (56.16)	0.077
Vitamin D insufficiency, N (%)	172 (37.53)	97 (40.42)	75 (34.25)	0.172
Vitamin D sufficiency, N (%)	49 (10.63)	28 (11.66)	21 (9.59)	0.471
TSH, mIU/L	3.33 (1.74–5.68)	3.23 (1.82–4.74)	3.52 (1.67–12.30)	0.010
T3, nmol/L	1.60 (1.30–1.80)	1.70 (1.50–1.90)	1.50 (1.20–1.80)	0.002
T4, nmol/L	105 (89–118)	106 (91–117.25)	103 (84.85–121)	0.203
fT4, pmol/L	12.10 (10.20–13.20)	12.10 (10.90–13.10)	11.90 (9.90–13.70)	0.104
TgAb, IU/mL	135 (36.40–422.40)	121.50 (26.40–321.30)	192 (49.30–596.05)	< 0.001
TPOAb, IU/mL	212 (27.60–652.90)	161.50 (17.40–529.75)	273 (66.40–945.50)	< 0.001
Thyroid volume, cm^3^	9.85 (7.30–13.91)	9.89 (7.72–13.26)	9.59 (6.82–14.90)	0.414
Number of symptoms, N	4 (1–7)	3 (1–6)	5 (2–8)	< 0.001
Systolic blood pressure, mmHg	120 (110–130)	115 (110–130)	120 (110–130)	0.279
Diastolic blood pressure, mmHg	70 (65–80)	70 (65–78.75)	70 (65–80)	0.396

ALL, all HT patients; MILD, HT patients that were euthyroid or in subclinical hypothyroidism; OVERT, HT patients that were in overt hypothyroidism or treated with levothyroxine (LT4) therapy. *Mann–Whitney test/chi-square test between MILD and OVERT; Q1-first quartile, Q3-third quartile.

Frequency of weekly consumption of 24 food groups in ALL, MILD, and OVERT HT patients is shown in Table 2. The most frequently consumed food groups are milk and milk products followed by vegetables (almost 10 times per week), whereas the least frequently consumed food include oily fish, white fish and seafood (less then once per week).

**Table 2:** Frequency of weekly consumption of 24 food groups in ALL, MILD, and OVERT patients with HT.

Food groups	ALL		MILD		OVERT		P*
	(*N* = 459)		(*N* = 240)		(*N* = 219)		
	Weekly intake (times/week)		Weekly intake (times/week)		Weekly intake (times/week)		
	Mean	SD	Mean	SD	Mean	SD	
Milk and milk products	9.83	5.52	10.00	5.74	9.63	5.28	0.497
Vegetables	9.53	5.96	9.13	5.68	9.99	6.25	0.144
Refined grains	6.12	3.66	6.21	3.76	6.01	3.57	0.566
Sweets	5.85	5.29	5.98	5.39	5.71	5.20	0.609
Coffee	5.18	2.90	5.00	2.97	5.41	2.80	0.154
Fruits	5.04	3.13	4.93	3.03	5.14	3.25	0.502
Plant oil	3.92	3.08	4.03	3.10	3.77	3.06	0.398
Olive oil	3.55	3.02	3.31	3.00	3.82	3.03	0.088
Potatoes	2.91	2.10	2.90	2.07	2.90	2.11	0.974
White meat	2.76	1.95	2.75	1.84	2.78	2.06	0.863
Processed meat	2.72	2.61	2.71	2.61	2.73	2.62	0.939
Tea	2.66	2.66	2.73	2.64	2.60	2.69	0.616
Red meat	2.54	2.10	2.31	1.99	2.80	2.20	0.017
Whole grains	1.76	2.26	1.78	2.24	1.74	2.30	0.844
Eggs	1.56	1.28	1.57	1.29	1.56	1.28	0.946
Non-alcoholic drinks	1.55	2.34	1.46	2.20	1.65	2.50	0.396
Nuts	1.48	1.90	1.41	1.83	1.56	1.99	0.443
Liquor	1.42	2.63	1.26	2.39	1.61	2.88	0.184
Jam, Marmalade	1.11	1.62	1.06	1.60	1.17	1.65	0.494
Salty snacks	0.94	1.49	0.99	1.57	0.88	1.39	0.481
Animal fat	0.88	1.51	0.73	1.21	1.04	1.77	0.043
Oily fish	0.79	0.96	0.74	0.81	0.85	1.10	0.247
White fish	0.69	0.87	0.65	0.84	0.73	0.90	0.327
Seafood	0.60	1.20	0.57	1.15	0.64	1.26	0.596

Weekly intake mean values are ordered by the frequency of consumption from the highest to the lowest in ALL HT patients. *t-test for independent samples between MILD and OVERT; SD, standard deviation.

We identified significant associations between vitamin D levels and the intake of three food groups in ALL and OVERT HT patients: (1) an inverse association between coffee consumption and vitamin D levels (ALL: *β* = −0.433, *p* = 0.005; OVERT: *β* = −0.62, *p* = 0.008); (2) an inverse association between sweets consumption and vitamin D levels (ALL: *β* = −0.195, *p* = 0.034; OVERT: *β* = −0.431, *p* = 0.006); (3) positive association between vegetable consumption and vitamin D levels (ALL: *β* = 0.182, *p* = 0.019; OVERT: *β* = 0.311, *p* = 0.009) (Table 3). We also identified three marginal positive associations with vitamin D: plant oil (ALL: *β* = 0.265, *p* = 0.060) milk and milk products (ALL: *β* = 0.145, *p* = 0.081) and non-alcoholic drinks (ALL: *β* = 0.333, *p* = 0.091; MILD: *β* = 0.529, *p* = 0.088).

**Table 3:** Association analysis results between vitamin D levels and consumption of 24 food groups in ALL, MILD, and OVERT patients with HT.

Food groups	ALL					MILD					OVERT				
	(*N* = 459)					(*N* = 240)					(*N* = 219)				
	*β*	SE	*p*	95.0% CI		*β*	SE	*p*	95.0% CI		*β*	SE	*p*	95.0% CI	
				Lower	Upper				Lower	Upper				Lower	Upper
Coffee	−0.433	0.154	0.005	−0.736	−0.131	−0.211	0.239	0.379	−0.685	0.262	−0.620	0.229	0.008	−1.074	−0.166
Vegetables	0.182	0.077	0.019	0.030	0.333	0.078	0.117	0.508	−0.154	0.309	0.311	0.117	0.009	0.079	0.543
Sweets	−0.195	0.092	0.034	−0.375	−0.015	−0.015	0.127	0.905	−0.266	0.236	−0.431	0.154	0.006	−0.735	−0.126
Plant oil	0.265	0.140	0.060	−0.011	0.540	0.169	0.206	0.413	−0.239	0.577	0.294	0.211	0.167	−0.125	0.713
Milk and milk products	0.145	0.083	0.081	−0.018	0.307	0.174	0.110	0.116	−0.044	0.392	0.035	0.143	0.806	−0.248	0.318
Non-alcoholic drinks	0.333	0.196	0.091	−0.053	0.720	0.529	0.308	0.088	−0.081	1.138	0.329	0.275	0.235	−0.217	0.875
Refined grains	−0.185	0.127	0.147	−0.436	0.065	−0.133	0.185	0.472	−0.499	0.232	−0.123	0.191	0.523	−0.502	0.257
Nuts	0.334	0.246	0.176	−0.151	0.818	0.606	0.380	0.113	−0.146	1.358	0.447	0.368	0.227	−0.282	1.175
Liquor	0.191	0.162	0.239	−0.128	0.511	0.042	0.259	0.871	−0.470	0.555	0.374	0.250	0.137	−0.121	0.870
White meat	−0.274	0.235	0.245	−0.736	0.189	0.357	0.402	0.377	−0.439	1.153	−0.544	0.335	0.107	−1.207	0.119
Jam, Marmalade	0.328	0.288	0.255	−0.239	0.895	0.489	0.449	0.278	−0.400	1.378	0.194	0.423	0.648	−0.645	1.032
Seafood	−0.541	0.529	0.307	−1.582	0.500	0.120	1.071	0.911	−1.999	2.239	−0.281	0.760	0.713	−1.788	1.227
Tea	−0.160	0.169	0.346	−0.494	0.174	−0.245	0.243	0.316	−0.727	0.236	0.029	0.252	0.909	−0.470	0.528
Oily fish	0.743	0.802	0.355	−0.835	2.322	2.067	1.347	0.127	−0.598	4.732	−0.077	1.113	0.945	−2.284	2.130
Processed meat	0.157	0.187	0.400	−0.210	0.524	0.146	0.272	0.593	−0.392	0.683	0.434	0.306	0.158	−0.172	1.040
Potatoes	0.186	0.225	0.409	−0.257	0.629	0.156	0.341	0.649	−0.519	0.831	0.239	0.319	0.456	−0.394	0.871
Salty snacks	0.232	0.310	0.454	−0.378	0.842	−0.169	0.457	0.712	−1.074	0.736	0.386	0.503	0.445	−0.612	1.383
Animal fat	−0.172	0.274	0.529	−0.712	0.367	−0.043	0.512	0.932	−1.056	0.969	−0.331	0.331	0.320	−0.988	0.326
Whole grains	−0.113	0.185	0.541	−0.478	0.251	−0.393	0.282	0.167	−0.951	0.166	0.148	0.299	0.622	−0.445	0.741
White fish	−0.398	0.656	0.545	−1.689	0.894	0.092	1.090	0.933	−2.065	2.248	−0.721	0.944	0.446	−2.593	1.150
Eggs	−0.174	0.302	0.564	−0.769	0.420	−0.013	0.441	0.977	−0.884	0.859	−0.428	0.508	0.401	−1.435	0.579
Olive oil	0.036	0.156	0.817	−0.271	0.343	0.047	0.232	0.840	−0.412	0.505	−0.163	0.229	0.478	−0.618	0.291
Red meat	−0.038	0.251	0.880	−0.533	0.457	−0.044	0.401	0.914	−0.837	0.750	−0.196	0.356	0.582	−0.901	0.509
Fruits	0.021	0.146	0.888	−0.267	0.308	0.179	0.217	0.410	−0.250	0.608	−0.136	0.207	0.513	−0.546	0.274

Linear regression model with vitamin D level as the dependent variable and 24 food groups as independent variables. Age, sex, score of physical activity during every day’s work, score of engagement in any sport activity, BMI, smoking status and seasonality of blood sampling were included as covariates in each model. In the group comprising ALL HT patients and subgroup of OVERT patients with HT, LT4 therapy status was used as an additional covariate. Food groups are ordered from the lowest to the highest value of *p* in ALL HT patients. Significant p-values are shaded in grey, whereas effect sizes of the significant food groups in ALL and OVERT patients are shown in bold. β, effect size; SE, standard error; CI, Confidence Interval for *β*.

## 4. Discussion

In this observational study, we analyzed the relationship between dietary patterns and vitamin D levels in patients with HT, the most common autoimmune disease today. The main findings of our study are observations of negative association between vitamin D levels and coffee and sweets consumption and positive association between vitamin D levels and vegetable consumption. Another important observation from our study is that effect sizes of all three observed associations were more prominent in HT patients with prolonged and more severe disease (OVERT).

Autoimmune diseases are often associated with reduced vitamin D levels (17). This is also true for our HT patients, since we observed low median vitamin D levels and high percentage of vitamin D deficiency, especially in the OVERT subgroup of HT patients (Table 1). Participants from our cohort live in the region of Split (Croatia) with the Mediterranean climate with more than 2,600 h of sunshine per year (equivalent to 108 days). Therefore, it is not easy to explain high proportion of vitamin insufficiency/deficiency in our participants since they live in a region with many sunny days. Although, we do not have information on daily sunlight exposure or outdoor activities of our participants, it appears that the lifestyle of our subjects is characterized by an increased indoor activities or office work. To overcome the problem of vitamin D deficiency, it is necessary to define the factors that affect vitamin D levels, especially those that are easily modifiable, such as those from the diet, which is the main focus of this paper.

We therefore aimed to test if there are, yet unknown, specific dietary patterns or types of food that are associated with decreased/increased vitamin D levels in ALL HT patients, and in subgroups of HT patients depending on disease severity (MILD and OVERT). We adjusted our analyses for all relevant “vitamin D modifying factors” (age, sex, BMI, smoking, physical activity and seasonality of blood withdraw) to decrease the impact of these factors on patient’s vitamin D levels and to minimize the heterogeneity between individuals. The main results of our study are observations of two inverse associations between vitamin D levels and consumptions of coffee and sweets (ALL and OVERT). We additionally detected one positive association between vitamin D levels and consumption of vegetables (ALL and OVERT) (Table 3). Importantly, we observe that effect sizes of all three associations are larger in HT patients with prolonged and more severe disease (OVERT) than in ALL patients (Table 3). Beside the marked thyroid dysfunction and a high rate of vitamin D deficiency, OVERT group of patients presents many distinct clinical features, especially when compared to patients in the beginning of disease (MILD). These include older age, higher BMI, higher thyroid antibodies and higher number of hypothyroidism symptoms (Table 1). It is well known that thyroid hormones affect basal metabolism and that patients with HT have many metabolic parameters disturbed, possibly including the vitamin D metabolic pathways (38, 39). Additionally, our group of patients with HT mainly consists of women (90.41% in OVERT) most of whom have vitamin D deficiency (Table 1). Therefore, beside appropriate therapy, adjusted diet and careful micronutrient intake can improve vitamin D levels of HT patients. In the next paragraphs we discuss our findings.

### 4.1. Inverse associations between food groups and vitamin D levels

#### 4.1.1. Coffee consumption and vitamin D

There are more than 1,000 different components in coffee. The best known are bioactive phenolic compounds such as caffeine, chlorogenic acids, cafestol, and kahweol, however, coffee also comprises various carbohydrates, lipids, nitrogenous components, vitamins, minerals, and alkaloids (40, 41). We observed relatively high frequency of coffee consumption in our cohort of HT patients, with an average of 5.4 times per week in ALL HT patients (Table 2). Many studies evaluated the impact of coffee on health outcomes. The largest study so far, an umbrella review of meta-analysis of coffee consumption and multiple health outcomes, concluded that coffee consumption is generally associated with beneficial effects on health, mirrored as reduction in all-cause mortality, cardiovascular mortality and total cancer (40). However, several harmful effects of coffee were also observed, namely, association with the risk of bone fractures in women (40). This last association links coffee intake with the bone metabolism, which is highly dependent on vitamin D levels.

Regarding association of coffee intake and vitamin D levels, two large-scale observational studies reported negative association between vitamin D levels and coffee intake (42, 43). Another study of 741 premenopausal women, which is more similar to ours with respect to the age and the sex of participants, found statistically significant association between higher intake of caffeine containing cola drinks, assessed through FFQ, and decreased vitamin D levels (44). Several other studies reported inconsistent findings regarding vitamin D levels and caffeine intake (45–47). Although there are contradictions in reports of coffee consumption and vitamin D levels, the results of our study are in line with the two biggest studies performed in healthy adult individuals (42, 43).

Our observation suggests that association between higher coffee consumption and decreased vitamin D levels may not only be the feature of general population but also of HT patients. It is therefore, important to understand the influence of coffee consumption on vitamin D levels, and subsequently on other linked metabolic parameters, such as those in bone metabolism. The data on this topic is very scarce and the causal relationship has yet to be investigated and explained. This leads us to an important message from our cross-sectional analysis, which is that observed inverse association between coffee consumption and vitamin D levels does not indicate a causal relationship between the two. For example, there may be another confounding phenotype that is associated with both phenotypes of interest (coffee and vitamin D), which is actually driving causality. Finally, it may be possible that individuals that regularly drink coffee also spend more time indoors than non-regular drinkers which may lead to residual confounding. However, given the plausible literature evidence of this inverse association, further research is highly required.

#### 4.1.2. Sweets and vitamin D

We also observed an inverse association between sweets intake and vitamin D levels (Table 3) in ALL and, especially, OVERT group. This food group is formed by combining the intake of several types of sweets with high sugar content (cakes, chocolate, cookies, bonbons). Our HT patients often consume sweets, almost six times per week, which puts this food group in the fourth place of the most consumed food groups (Table 2).

Several studies have also previously observed a negative association between consumption of sweets (or sugar) and vitamin D levels. For example, an inverse association between sugar intake, assessed by FFQ, and vitamin D levels was observed in 32 participants from 13 to 25 years of age (48). Similarly, a study of 129 healthy Argentinian boys observed an inverse association between vitamin D levels and plasma glucose concentration (49). Finally, consumption of sugar-sweetened non-alcoholic beverages showed negative association with vitamin D levels (44). Taken altogether, observational studies consistently point to an inverse relationship between high sugar intake and low vitamin D levels. However, there are no clear functional explanations for observed associations. Of relevance, vitamin D is involved in glucose homeostasis/metabolism (50–52), regulation of insulin secretion and insulin resistance (50, 52, 53). There are also number of studies that report an inverse association between vitamin D and metabolic parameters such as fasting glucose or insulin resistance (54–57).

Another thing to bear in mind is that there is a link between coffee (our main result) and sugar consumption as sugar is often added to coffee to soften its bitterness. Therefore, sugar added to coffee may be an additional factor, to the regular coffee ingredients, that may negatively affect vitamin D levels.

### 4.2. Positive associations between food groups and vitamin D levels

#### 4.2.1. Vegetables and vitamin D

We observed significant positive association between vegetable consumption and vitamin D levels in ALL and OVERT patients. Vegetables are one of the most frequently consumed food groups in our cohort with an average of 9.53 times per week in ALL HT patients (Table 2). Several studies have also observed positive associations between plant-based food and vitamin D levels, such as the study of 73 people from Australia, explaining this by the beneficial impact of a wide range of plant micronutrients and phytochemicals on vitamin D levels (58). Another positive correlation between “fruits and vegetables” diet and vitamin D, assessed through FFQ, was shown in 4372 participants from Switzerland (59). Yet another study observed significant correlation between vegetable diet and vitamin D levels suggesting that diet rich in vegetables may have a role in the maintenance of vitamin D levels in children (60). Additionally, an adherence to healthy Mediterranean diet, that includes frequent consumption of vegetables and dairy products (61) was positively associated with vitamin D levels (62). Similarly, a study in Uruguayan population found that higher intake of vegetables, nuts and fish was associated with lower risk of vitamin D deficiency (63).

Plant food is generally thought to be poor with vitamin D content (64), however, recent data suggest that plants may also be the source of vitamin D, especially vitamin D3 and its metabolites (65). Nevertheless, common anti-inflammatory roles of vitamin D and nutrients from vegetables may be a crosslink for the observed association and deserve further investigation.

#### 4.2.2. Plant oil and vitamin D

Consumption of plant oil was found to be marginally significant in ALL patients with HT. Positive association may be explained by usual fortification of a plant oils with vitamin D (66). The most frequently consumed plant oil in Croatia is a sunflower oil that is typically fortified with vitamin D.

#### 4.2.3. Milk products and vitamin D

We also found marginally significant positive association between consumption of milk and dairy products with vitamin D levels in ALL patient with HT. Milk/milk products is also one of the most frequently consumed food groups in our cohort with an average of 9.83 times per week in ALL group. Since our FFQ did not differentiate the type of milk (unfortified or fortified with vitamin D) we can conclude that observed association between milk/milk products and vitamin D levels can be, at least partially, explained by vitamin D fortification.

#### 4.2.4. Non-alcoholic drinks and vitamin D

We identified marginally significant positive association between non-alcoholic drinks intake and vitamin D levels in ALL and MILD. Although non-alcoholic drinks are usually sugar-sweetened and generally show a negative association with vitamin D levels (52, 67), our opposite result may be explained by a common intake of a specific non-alcoholic national drink, called Cedevita, in our population. It is a naturally fruity flavored multivitamin drink which is a source of 9 vitamins (vitamin C, E and B complex: B1, B2, B3, B5, B6, B9, and B12). We have already observed beneficial effects of this drink on general health in our previous study (10). However, we have to stay cautious with interpretation of our results, as there is no prior evidence of the beneficial effect of non-alcoholic drinks to vitamin D levels.

### 4.3. Absence of associations between vitamin D levels and vitamin D rich food

We did not detect associations of vitamin D levels with the two well-known food sources of vitamin D: oily-fish and eggs. The lack of association can be explained by the very low frequency of oily-fish consumption, which is less than once per week (0.79 times per week in ALL HT patients). The same applies to the consumption of eggs, which are consumed on average 1.56 times per week in ALL HT patients (Table 2). Therefore, the contribution of these two foods to the total levels of vitamin D in our HT patients is low. In addition, although our participants come from the Mediterranean region of Croatia, only 8.6% of them strictly adhere to the Mediterranean diet (manuscript under preparation), which means that vitamin D levels of the vast majority of our patients are not increased by the known vitamin D rich food. Beside vitamin-D rich food, the other main sources of vitamin D are fortified foods, mainly dairy products and supplements (64, 68, 69). According to the current data, fortification of dairy products with vitamin D is carried out in several countries: USA, Canada, Sweden, Norway, and Finland. Other countries, such as Croatia, do not have systematic vitamin D-milk fortification (23). Randomized control studies and observational studies have shown positive effects of fortified foods on serum vitamin D levels, and the association was stronger in countries with national vitamin D fortification policies (23, 32).

### 4.4. Limits and advantages

A limitation of our research is that our FFQ was not designed to collect quantitative data of food intake, therefore we could not calculate nutritional composition of consumed food. Another limitation is that our observational study was not designed to determine the causality between dietary habits and vitamin D levels. The causality between observed associations need to be further investigated using various analytical strategies. One of them, Mendelian randomization methods, may provide reliable evidence of causality before embarking on randomized control trials (70).

The biggest advantage of our study is that we used a large cohort of stringently diagnosed HT patients recruited in clinical settings with precisely defined two subgroups stratified by severity of disease. To our knowledge, this is the first study that analyzed association of dietary habits with vitamin D levels in patients with HT.

### 4.5. Conclusion

Insufficient levels of vitamin D and high proportions of vitamin D deficiencies are global health burden for all, healthy and diseased individuals. Patients with HT are not exception to this problem, mainly those in more severe stage of disease. Practical solutions for increasing vitamin D levels include higher exposure to sun light, intake of vitamin D supplementation and vitamin D rich or fortified food. Additionally, avoidance of factors that may negatively affect vitamin D blood levels may alleviate the problem of vitamin D deficiency (71, 72). Non-pharmacological factors that may affect vitamin D levels, such as those from food, are becoming increasingly important, as they are cheaper and easier to modify. For example, consumption of foods that show evidence of negative association with vitamin D levels, such as coffee and sweets, should be avoided, at least during winter–spring season. Functional and causal relationships of observed associations are not known and need to be further investigated to ultimately provide guidelines regarding coffee/sugar intake and vitamin D status. The results of our study show that food factors may have greater impact on vitamin D levels in OVERT HT patients in more severe stages of disease.

## Data availability statement

The raw data supporting the conclusions of this article will be made available by the authors, without undue reservation.

## Ethics statement

Ethics Committee from the University of Split School of Medicine (Classification no. 003–08/14–03/0001 and Registry no. 2181–198–03-04-14-0028; Classification no. 003–08/19–03/0003 and Reg. no. 2181–198–03-04-19-0019) and the Ethics Committee from the University Hospital of Split (Classification no. 530–02/13–01/11; Registry no. 2181–147-01/06/J.B.-14–2; Classification no. 500–03/18–01/80 and Reg. no. 2181–147-01/06/M.S.-18–2) approved this research. The patients/participants provided their written informed consent to participate in this study.

## Author contributions

VB and DK conceived the study idea and drafted the manuscript. DK, AB, AP, and VB formed the biobank of patients with Hashimoto’s thyroiditis (CROHT). MC and AB performed the measurements and diagnosis of HT. DK performed the statistical analysis. VŠ and AP reviewed the manuscript. All authors have read and agreed to the final version of the manuscript.

## Funding

This formation of the CROHT biobank was supported by the Croatian Science Foundation under the project “Genome-wide association analysis of Hashimoto’s thyroiditis” (grant no. 4950). Vitamin D measurements were obtained under the HAZU Foundation project “Analysis of the role of vitamin D with the presence and clinical manifestation of Hashimoto’s thyroiditis.”

## Conflict of interest

The authors declare that the research was conducted in the absence of any commercial or financial relationships that could be construed as a potential conflict of interest.

## Publisher’s note

All claims expressed in this article are solely those of the authors and do not necessarily represent those of their affiliated organizations, or those of the publisher, the editors and the reviewers. Any product that may be evaluated in this article, or claim that may be made by its manufacturer, is not guaranteed or endorsed by the publisher.

## Figures and Tables

**Table:** 

AITD	Autoimmune thyroid disorders
ALL	Group of all patients with HT
CROHT	Croatian Biobank of patients with Hashimoto’s thyroiditis
ETA	European Thyroid Association
FFQ	Food frequency questionnaire
HT	Hashimoto’s thyroiditis
LT4	Levothyroxine therapy
MUFA	Monounsaturated fatty acids
MILD	HT patients that were euthyroid or in subclinical hypothyroidism
OVERT	HT patients that were in overt hypothyroidism or treated with levothyroxine therapy
SE	Standard error
SD	Standard deviation
TgAb	Antibodies against thyroglobulin
TPOAb	Antibodies against thyroid peroxidase
TSH	Thyroid-stimulating hormone
T3	Triiodothyronine
T4	Thyroxine
fT4	Free thyroxine
VDR	Vitamin D receptor
1.25(OH)2D	Calcitriol
25(OH)D	Calcidiol or 25-hydroxyvitamin D
